# Simultaneous closed rupture of flexor digitorum superficialis and flexor digitorum profundus tendons in the middle finger: a case report

**DOI:** 10.3109/23320885.2014.973500

**Published:** 2015-01-06

**Authors:** Robert W. Jordan, Naeil Lotfi, Gunaratnam Shyamalan

**Affiliations:** ^a^ Trauma & Orthopaedic Department, Birmingham Heartlands Hospital, Birmingham, UK

**Keywords:** Flexor tendon, hand, hand finger, trauma

## Abstract

A 20-year-old man suffered a closed rupture of both flexor tendons in the middle finger while playing rugby. Primary repair of the flexor digitorum profundus and excision of the flexor digitorum superficialis was performed. At follow up he reported a Disabilities of the Arm, Shoulder and Hand score of 0 and unrestricted return to activities.

## Introduction

Closed tendon ruptures are rare as they represent the strongest link in the musculotendinous chain, with injury to the musculotendinous junction, muscle or its insertion being more common [1]. A spontaneous tendon rupture occurs in the absence of intrinsic or extrinsic pathologic processes [1]. This process often arises in degenerative tendons such as in rheumatoid arthritis, gout or diabetes mellitus. The use of certain medications such as fluoroquinolone antibiotics has been associated with increased risk of tendinopathy and rupture. Spontaneous rupture with no obvious pathology seems to result from repetitive trauma.

The term ‘Jersey Finger’ has commonly been used to describe an isolated rupture of the flexor digitorum profundus (FDP) [2]. This typically occurs when a sportsperson grasps an opponent’s jersey who pulls away causing forcible extension of the flexed finger. Closed rupture of both flexor tendons of the same digit is rare; only a handful of cases have been reported in the literature [1, 3, 4, 5, 6, 7, 8]. These reports describe various mechanisms of injury: hyperextension [1, 3, 4], forced flexion [1], direct blow [7] and gradual rupture following repetitive actions [5, 8]. The diagnosis is clinical but can be supported by both ultrasound and magnetic resonance imaging which can provide additional information on tendon integrity, location of injury and the distance between tendon ends [9, 10]. We present a case of closed injury to both flexor digitorum superficialis (FDS) and FDP that was managed by primary repair of the FDP and excision of` the FDS.

## Case report

A 20-year-old, left-handed man was playing rugby when he tackled another player, gripping his rugby shirt. Immediately he suffered pain on his left middle finger but was able to complete the remainder of the game. Afterwards his finger had swollen and he was unable to bend it fully. He presented to his local emergency department where the extent of the injury was not recognised; he was provided with neighbour strapping and given follow up in his local orthopaedic clinic for 1 week after the injury. On examination at orthopaedic follow up, he was unable to flex the distal interphalangeal joint (DIPJ) and had very weak flexion at the proximal interphalangeal joint (PIPJ). Passive movement was maintained at the DIPJ but he had a restricted passive range of motion at the PIPJ. An initial diagnosis of a closed rupture of the FDP tendon was made and the patient referred to a hand specialist who counselled the patient for exploration and tendon repair or reconstruction.

**Figure 1.**
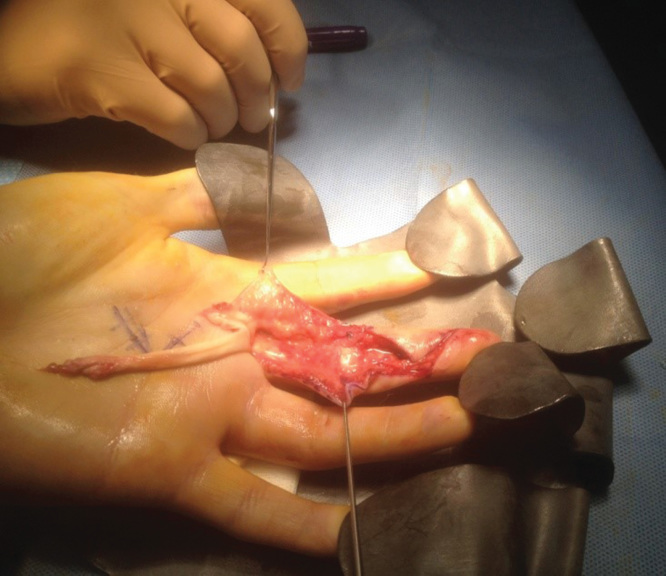
**Intra-operative image demonstrating ruptures of both flexor digitorum profundus and flexor digitorum superficialis tendons.**

Surgical exploration was performed on day 14 after his injury and under anaesthesia tenodesis revealed no flexion at the PIPJ or DIPJ. Initially, a Brunner’s incision was performed opening the A5 pulley where a rupture of the FDP tendon was identified. It was not possible to milk the retracted FDP tendon so the initial incision had to be extended proximally to the A1 pulley and the FDP was identified and delivered. However, a continued restricted movement at the PIPJ led to opening of the A3 pulley where a complete rupture of the FDS tendon was revealed (Figure 1). The FDS tendon was not repaired as it was found to be swollen and the vinculae damaged; instead, the FDS was trimmed to facilitate repair of the FDP. The FDP tendon was threaded back through the residual pulleys and finally secured to the distal phalanx using a dorsal pull through technique.

**Figure 2.**
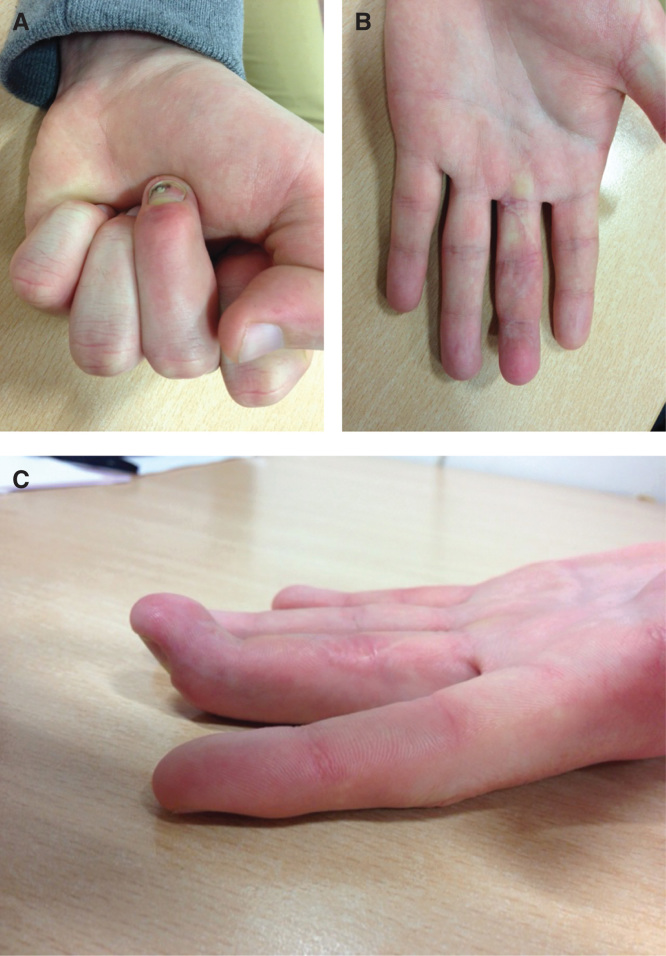
**2*a–c*. Illustration of range of movement at 4 months post-surgery.**

The patient was splinted in the Edinburgh position and was commenced on an active range of motion protected with a dorsal splint. Unprotected movement of the finger was allowed from 8 weeks. He was reviewed in the outpatient clinic at 4 months where he had full movement at the PIPJ and an arc of 20° to 70° of flexion at the DIPJ (Figures 2*a–c*). The patient reported Disabilities of the Arm, Shoulder and Hand score of 0 and he had successfully returned to work and sport.

## Discussion

**Figure 3.**
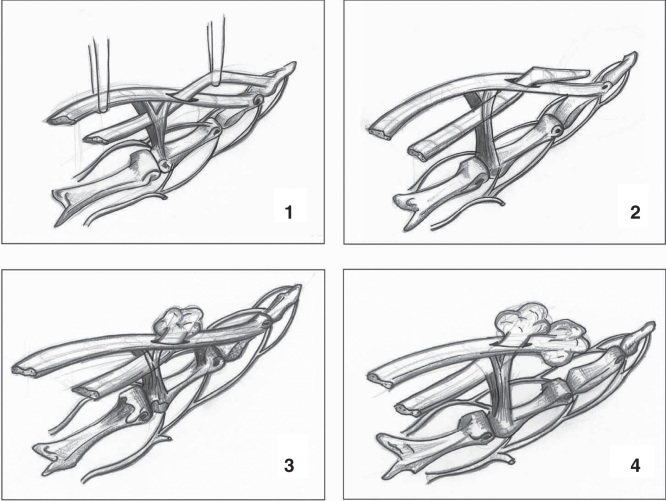
**Illustration of proposed mechanism: 1) demonstration of normal anatomy; 2) flexor digitorum profundus (FDP) tendon ruptures first, 3) as FDP retracts, it becomes trapped in the decussation of the flexor digitorum superficialis (FDS) at the level of Camper’s chiasm; 4) continued forced extension against the contracted FDS/FDP tendons results in FDS tendon rupture.**

Closed rupture of both flexor tendons in one finger are rare, with the mechanism of injury varying in the reported literature [1, 3, 4, 5, 6, 7, 8]. We report a case of closed rupture of FDP and FDS tendons following forced extension of maximally contracted flexor muscles. The authors propose that the FDP tendon ruptured first as usually occurs during a ‘Jersey Finger’ [2]; the retracted FDP then became trapped in the decussation of the FDS at the level of Camper’s chiasm. Continued forced extension against the contracted FDS/FDP tendons resulted in the FDS tendon rupture. The ruptured tendon ends became lodged under the A3 pulley stopping further retraction, as illustrated in Figure 3. However, this is only a proposal and the exact mechanism is unknown.

The failure to recognise the injury at presentation delayed surgical exploration and led to inadequate immobilisation of the finger. Whether the inability to immobilise the finger contributed to the subsequent FDS rupture or whether this had already occurred is unknown. The small amount of flexion at the PIPJ at initial clinical examination makes this a possible explanation and in future cases immobilisation of the finger is recommended until surgical intervention. This case report should raise awareness that injury to both tendons should be sought if any restriction of movement is demonstrated at the PIPJ. Although imaging has been shown to provide additional information in complex tendon injuries [9, 10], the decision to proceed without imaging in this case was made to avoid any further delay to surgery which would have impeded repair.

This combination of injuries to FDS and FDP tendons are more commonly encountered following open injury where they provide an equally stern surgical challenge [11]. These injuries are at high risk of adhesion formation due to the intimacy of the tendon relationship at this level. The aim of surgery is to reduce iatrogenic trauma, provide a repair of sufficient strength to allow for early rehabilitation and achieve a smooth repair site to minimise work during flexion [11]. Our patient was managed with excision of the FDS tendon and repair of FDP with predictable results. Out of the available case presentations, three cases were treated with tendon transfer [3, 5, 8], whereas two cases were treated with FDP repair with trimming/resection of the FDS tendon [4, 7].

The decision whether to repair either FDS and FDP tendons or only the FDP is controversial. Repair of both tendons has been advocated to improve functional recovery and preserve the blood supply to the FDP through the vincula system [12, 13]. However, difficulty may be encountered in repairing tendons if the tendon ends are swollen and frayed, which is common in cases of delayed repair. This increases the risk of tendon adhesion, and excision of FDS tendon may avoid this complication. Tang performed a randomised controlled trial comparing these two techniques; the author reported similar motion between the two groups but a higher rate of complication following repair of both tendons [14]. In the case reported, the decision to repair only the FDP was made as the FDS tendon was found to be swollen and repairing both tendons while maintaining the A2 pulley would not be feasible. The damage to the vinculae was considered an additional risk for adhesion formation and contributed to decision making.

Due to the infrequency of the condition and lack of a penetrating wound, there may be a delay to diagnosis which can impact on treatment decision making. Pike and Gelberman suggest that primary repair is not feasible if the delay is over 4 weeks with two-stage reconstruction required in these situations [11]. In the case reported, delayed repair was facilitated by the ruptured tendons becoming trapped at the A3 pulley. In these cases imaging could be crucial in determining at which level the tendons have retracted and help decide whether delayed repair is feasible.
